# Microsatellite evidence of dispersal mechanism of red swamp crayfish (*Procambarus clarkii*) in the Pearl River basin and implications for its management

**DOI:** 10.1038/s41598-017-08552-3

**Published:** 2017-08-15

**Authors:** Jinlong Huang, Shaoqing Tang, Fengjin Cai, Yanfang Lin, Zhengjun Wu

**Affiliations:** Guangxi Key Laboratory of Rare and Endangered Animal Ecology, College of Life Science, Guangxi Normal University, Guilin, 541004 China

## Abstract

Discerning the dispersal patterns of invasive species is critically important for the design of effective management strategies and the development of appropriate theoretical models predicting the spatial expansion of introduced populations. Post-introduction dispersal may occur naturally or via human transport, but for many organisms, assessing the relative contribution of each of these factors is difficult using traditional methods. Here, we explored the genetic patterns associated with the spread of red swamp crayfish (*Procambarus clarkii*) among 21 populations in the Pearl River basin and 2 peripheral populations in the Yangtze River basin. We found the genetic diversity of *P. clarkii* in the Pearl River basin was somewhat lower than in the Yangtze River basin. We also found (1) there was significant genetic differentiation between populations, (2) genetic differentiation was not related to geographic distance (i.e., isolation by distance), and (3) a Bayesian assignment analysis revealed three distinct genetic clusters and genetic admixture. Our results therefore provide evidence that human-mediated multiple introductions occurred in the Pearl River basin. Anthropogenic activities such as commercial transportation were likely responsible for the long-distance dispersal of *P. clarkii*. This study provides useful information for developing management strategies.

## Introduction

Bio-invasions are considered a result of global trade, human transport and climate change^1, 2^ and have become a world-wide problem threatening global biodiversity, disrupting key biological interactions and causing massive economic loss^3^. There is an increasing need to take action to control biological invasions and thus mitigate their impacts on biodiversity, ecosystem services, and human activities. Prevention is generally far more cost-effective and environmentally desirable than post-introduction measures such as eradication or long-term containment. Before implementing appropriate prevention strategies, it is necessary to collect detailed information about the dispersal pattern and vectors of an invasion. However, traditional (mark-recapture method) quantification of actual dispersal patterns of invasive species is a huge challenge and cannot provide effective information on invasive species population dynamics^4^.

Fortunately, molecular systematics and population genetics have rendered new and exciting tools to better our understanding of population dynamics during biological invasions^5^. Genetic tools provide information on the pathway(s) of an invasion, estimate inoculum size, determine whether an invasion consists of one or more introduction events, and show whether admixtures occur in the introduced range^6^. A genetic approach to the management of invasive species is gathering momentum. The information gleaned from this analysis of gene flow and population structure has broad implications for quarantine, trade and management of *Bactrocera correcta* ^7^, *Bemisia tabaci* biotype B^8^, and *Vulpes vulpes* ^9^. Coupled with ecological information on dispersal and/or colonization mechanisms and life-history characteristics, population genetics analyses provide a strong basis to formulate management strategies^10^.

The red swamp crayfish (*Procambarus clarkii*) is a highly successful invader originating from the south-central United States and northeastern Mexico^11^. According to records, *P. clarkii* was first intentionally introduced to Nanjing, China, from Japan in 1929^12^ and subsequently introduced into many provinces in China. Presently, *P. clarkii* was detected in a new watershed in the Pearl River basin. Successful establishment of *P. clarkii* populations has been facilitated by their high environmental tolerance, rapid population growth rate and superior competitive ability^13, 14^. This invasive crayfish is a polytrophic keystone species that can exert multiple pressures on ecosystems^15^. Most studies have focused on the effects of the decline in macrophytes and predation on several species (amphibians, molluscs, and macroinvertebrates), highlighting how this biodiversity loss leads to unbalanced food chains^15–17^. Furthermore, *P. clarkii* is a host and vector for obligate parasites such as *Aphanomyces astaci* and spreads contagious and lethal disease to the indigenous crayfish *Austropotamobius pallipes* ^18^. Thus, expansion and establishment of *P. clarkii* in new areas may convey considerable risks for introducing and transmitting this disease and obligate parasites to native species.

To minimize the ecological consequences, there is a need to manage the expansion of *P. clarkii*. It has been reported that it can disperse terrestrially between adjacent, or nearly adjacent, environments^19^. However, without human assistance, it will not invade such large areas in China. The commodity property of *P. clarkii* as an aquatic product increased the large-scale invasion to a large extent^20^. Previous works have demonstrated the initial entry point, genetic structure of *P. clarkii* and dispersal pattern mediated by human activities in the Yangtze River basin^12, 21^. However, we were not aware of reports of the expansion of *P. clarkii* across different geographic boundaries in the Pearl River basin.

In this study, we aimed to investigate population genetics patterns associated with the spread of *P. clarkii* in the Pearl River basin and proposed some management opinions. Genetic analyses were based on data from seven highly polymorphic microsatellite loci. We utilized both population- and individual-level assessments of genetic connectivity between sample sites to infer dispersal patterns associated with multiple potential introductions acting on different spatial scales.

## Materials and Methods

### Sample collection

In 2011–2012, *P. clarkii* specimens were collected from twenty-three sites in ditches, ponds and rivers, including twenty-one populations in the Pearl River basin (including fifteen populations in Guangxi province, five populations in Guangdong province and one in Hunan province) and two outside of the Pearl River basin (Table S1). According to records, Nanjing in Jiangsu province was the first place where *P. clarkii* was introduced^12^. Therefore, we collected *P. clarkii* specimens in Nanjing as a peripheral population. Moreover, we sampled opportunistically from Hunan province as another peripheral population, with the intent of assessing patterns in population structure across the river basin. During our investigation, we found there no distribution of *P. clarkii* in the upstream and East River areas of the Pearl River basin. Most populations were distributed in the midstream and downstream areas of the Pearl River basin. We collected *P. clarkii* specimens from wild natural water. The minimum geographical distance between adjacent sample sites was more than 20 km. A total of 904 adult individuals were collected. From each population, the sample number ranged from 24 to 45 individuals (see details in Table S1). Abdominal muscle tissues were collected from each individual and stored in absolute ethanol for subsequent experiments.

### DNA extraction and microsatellite genotyping

We extracted genomic DNA from ~0.1 g of muscle tissue using phenol-chloroform, and total DNA was preserved at −20 °C. Seven polymorphic microsatellite loci (PclG-02, -07, -13, -16, -17, -27, -28) were amplified as previously described^22^. We performed PCR on a Bio-Rad machine in a 15 μL reaction volume containing 1 μL PCR buffer, 2.5 mM MgCl_2_, 0.2 mM dNTPs, 0.2 μM of each microsatellite primer, 1.0 U rTaq polymerase (TaKaRa), 1.0 μL template DNA, and deionized water to a final volume of 15 μL. PCR cycling conditions were: 94 °C for 2 min; followed by 35 cycles of 94 °C for 30 s, 55 °C for 30 s and 72 °C for 40 s; and final extension at 72 °C for 10 min. Amplified products were separated by 6% denaturing polyacrylamide gel electrophoresis and stained with AgNO_3_.

### Genetic data analysis

We performed several tests on microsatellite loci to check scoring errors and violations of assumptions in our statistical analyses. We tested deviations from Hardy-Weinberg equilibrium (HWE) for each locus in each population with 10,000 dememorizations, 5,000 batches, and 5,000 iterations per batch using Genepop on the Web^23^. Linkage disequilibrium for each pair of loci in each population was also tested using the same software. We used Bonferroni’s correction^24^ to evaluate the significance of deviations from HWE or linkage disequilibrium. In addition, we used Micro-Checker software^25^ to test for the presence of null alleles. Finally, we found that the genotyping error rate was 0.087% in all loci of total populations. Therefore, we are confident that our genetic data were sufficiently robust to analyse genetic diversity and population structure.

We calculated the average number of alleles, observed heterozygosity (*H*o), and expected heterozygosity (*H*e) for each population using GenAlEx version 6.5 software^26^. To assess differentiation among populations and analyse the genetic relationships among populations, pairwise *F*_ST_ values and pairwise genetic distances were calculated using the same software, respectively. Neighbour-joining trees were constructed in Mega v6.0^27^.

To detect bottlenecks, a test of heterozygosity excess was performed using the program Bottleneck 1.2.02^28^. Bottleneck is based on the premise that the number of alleles decreases faster than gene diversity (i.e., expected heterozygosity) when populations experience a bottleneck^29^. We tested bottlenecks using both the stepwise-mutation (SMM) and two-phased (TPM) models with 10,000 replicates. Variance for TPM was set to 30, and the proportion of SMM in TPM was set to 80%. In our models, we used the Wilcoxon sign rank test to establish whether the number of loci showing heterozygosity excess was significantly greater than expected in populations at equilibrium. A mode shift in allele frequency distribution was also used as an indicator of a population bottleneck^30^.

We used analysis of molecular variance (AMOVA) to partition genetic variation within and among populations using Arlequin v3.5 software^31^. In AMOVA, total variance is partitioned into separate components, each of which describes the proportion of the total variance at distinct hierarchical levels. We tested for a relationship between geographic distance and genetic distance using Mantel tests on the Isolation By Distance (IBD) Web Service v3.23^32^. The geographic distances between populations were calculated from the coordinates of each sample sites using gpsCalc. We conducted a IBD analyse for overall population test. In order to more precisely illustrate IBD models of populations along rivers, we conducted separate IBD analyses for sample sites: (1) populations Xing’an (XA), Lingchuan (LC), Lingui (LG), Yangshuo (YS), Lipu (LP) and Zhaoping (ZP) along the Guijiang River and (2) populations Chenzhou (CZ), Yingde (YD), Qingyuan (QY), Sihui (SH), Sanshui (SS) and Gaoming GM) along the North River. The distances between populations along Guijiang River and the North River were used river kilometre distances.

Finally, we evaluated the genetic structure using Bayesian assignment analysis in Structure v2.3.4^33^. We assessed the likelihood of models with the number of clusters ranging from *K* = 1 to *K* = 11. We performed 20 independent runs for each value of *K* with Markov Chain Monte Carlo (MCMC) using 1,000,000 replicates and a length of burn-in period of 100,000 replicates. Based on likelihood scores among runs, Δ*K* of the corresponding *K* values were calculated according to an algorithm described by Evanno *et al*.^34^. We chose the model with the highest Δ*K* as the best one representing the true underlying genetic structure. The corresponding value of *K* was determined to be the best estimate of the number of clusters in the full data set.

## Results

We genotyped a total of 904 individuals from 23 populations with seven polymorphic microsatellite loci. Microsatellite loci were found to be robust in tests of HWE and linkage disequilibrium. The great majority of loci were found to be in HWE after sequential rounds of Bonferroni’s correction, except for locus PclG-16 in the Shatang (ST) population. No loci exhibited significant linkage disequilibrium after sequential Bonferroni’s correction.

We found a total of 39 alleles at the seven microsatellite loci in the 23 populations. Locus PclG-28 had the most alleles (nine) and locus PclG-13 had the least (two). The average number of alleles per locus in the population ranged from 2.57 to 4.14 (Table S1). The average number of effective alleles ranged from 1.73 to 2.75. *H*o ranged from 0.25 to 0.60 and *H*e ranged from 0.289 to 0.598. The Nanjing (NJ) population had the most genetic diversity among populations. Except for two peripheral populations, the Sihui (SH) population had the most genetic diversity among populations in the Pearl River basin.

AMOVA showed that the genetic variation within populations contributed more to genetic diversity than between populations (Table 1); 37% of genetic variation was partitioned among populations in the AMOVA and 63% of genetic variation occurred within populations. The average *F*_ST_ was 0.13. The greatest differentiation occurred between the Xiangzhou (XZ) population and the Fuchuan (FC) population (*F*_ST_ = 0.41) and the smallest was between the Lingui (LG) population and the Yongfu (YF) population (*F*_ST_ = 0.01) (Table S2).

**Table 1:** Analysis of molecular variance (AMOVA) for 23 population structures of *P. clarkii*.

Source of variation	*d. f*.	SS	MS	Est. Var	%
Among populations	22	2746.48	124.84	3.05	37
Within populations	881	4587.59	5.21	5.21	63
Total	903	7334.07		8.26	100

TPM and SMM models were applied to test whether the microsatellites displayed a departure from the mutation drift equilibrium. Using the TPM model, Wilcoxon’s test revealed that thirteen populations might experience bottleneck (*P* < 0.05 or *P* < 0.01 respectively). Using the SMM model, it is likely that there was a bottleneck in six populations (*P* < 0.05 or *P* < 0.01) (Table 2).

**Table 2:** *P*-value for Wilcoxon’s test for heterozygosity excess conducted in Bottleneck for twenty-three *P. clarkii* populations.

Population (abbreviation)	*P*-value of Wilcoxon test		Population (abbreviation)	*P*-value of Wilcoxon test	
	T.P.M.	S.M.M.		T.P.M.	S.M.M.
Lipu (LP)	0.0078^**^	0.0547	Zhaoping (ZP)	0.0547	0.1484
Lingui (LG)	0.0078^**^	0.0547	Zhongshan (ZS)	0.1484	0.2891
Yongfu (YF)	0.0039^**^	0.0273^*^	Fuchuan (FC)	0.5000	0.6875
Yangshuo (YS)	0.0039^**^	0.0273^*^	Yingde (YD)	0.0039^**^	0.0039^**^
Xing’an (XA)	0.2188	0.4219	Qingyuan (QY)	0.0078^**^	0.0391^*^
Lingchuan (LC)	0.0547	0.2891	Sihui (SH)	0.0078^**^	0.1484
Shatang (ST)	0.0078^**^	0.1484	Gaoming GM)	0.5938	0.9453
Luzhai (LZ)	0.2344	0.5938	Sanshui (SS)	0.0195^*^	0.1484
Liujiang (LJ)	0.0039^**^	0.0078^**^	Chenzhou (CZ)	0.0391^*^	0.1875
Xiangzhou (XZ)	0.3438	0.7109	Yueyang (YY)	0.1875	0.4688
Guigang (GG)	0.0078^**^	0.0078^**^	Nanjing (NJ)	0.0273^*^	0.1484
Babu (BB)	0.1875	0.2891			

TPM: two-phased model of mutation, SMM: stepwise mutation model, *P < 0.05, **P < 0.01.

**Figure 1:**
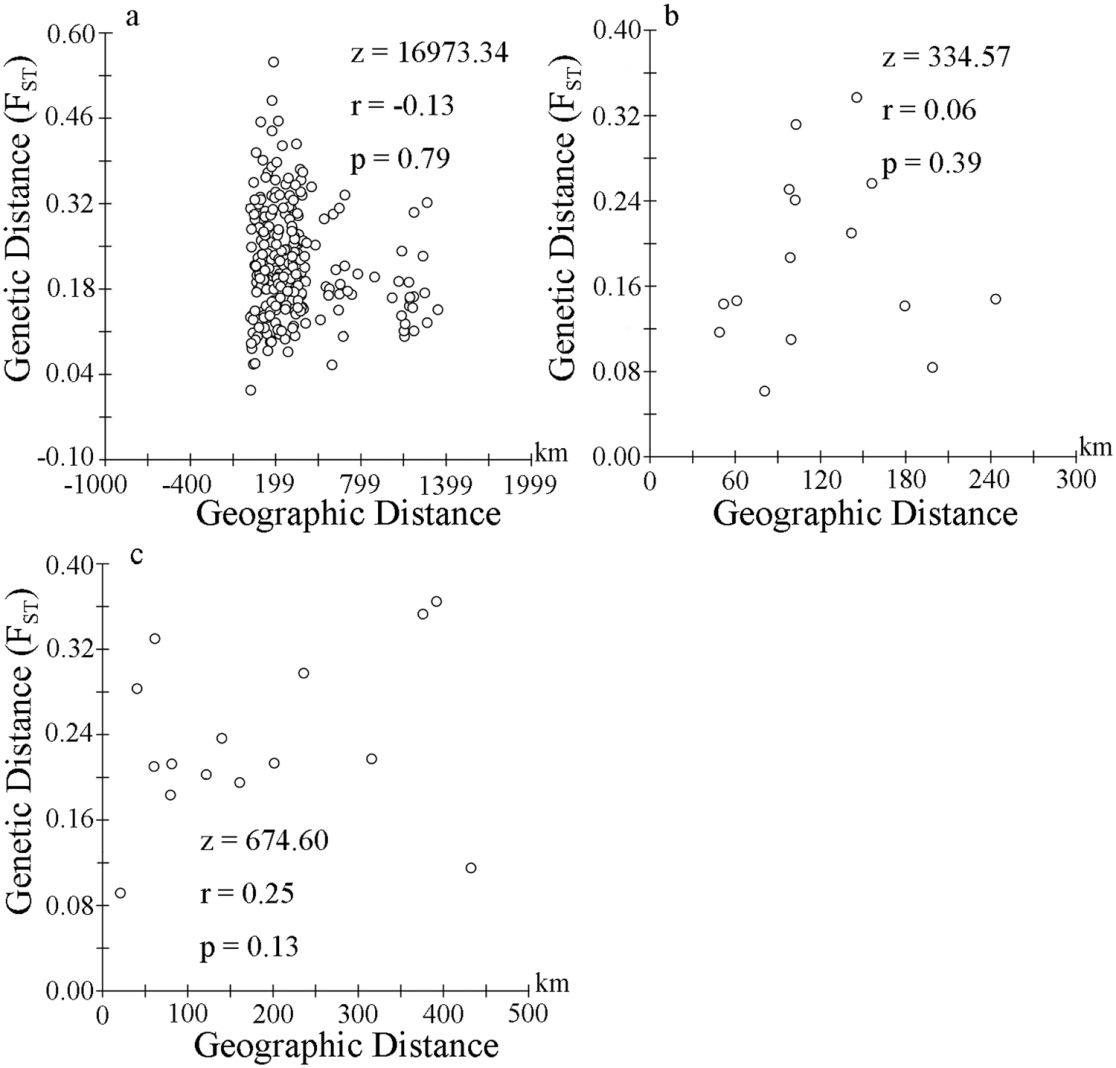
Relationship between genetic distance (*F*_ST_) and geographic distance for (**a**) plot of overall populations in the Mantel test, (**b**) plot of Guijiang River populations (XA, LC, LG, YS, LP, ZP) in the Mantel test, (**c**) plot of North River populations (YD, QY, SH, SS, GM, CZ) in the Mantel test.

A Mantel test of IBD revealed no relationship between genetic distance and geographic distance among all populations (Fig. 1a). We found a pattern of IBD along populations in the Guijiang River and North River, respectively, but it was not significant (Fig. 1b,c).

**Figure 2:**
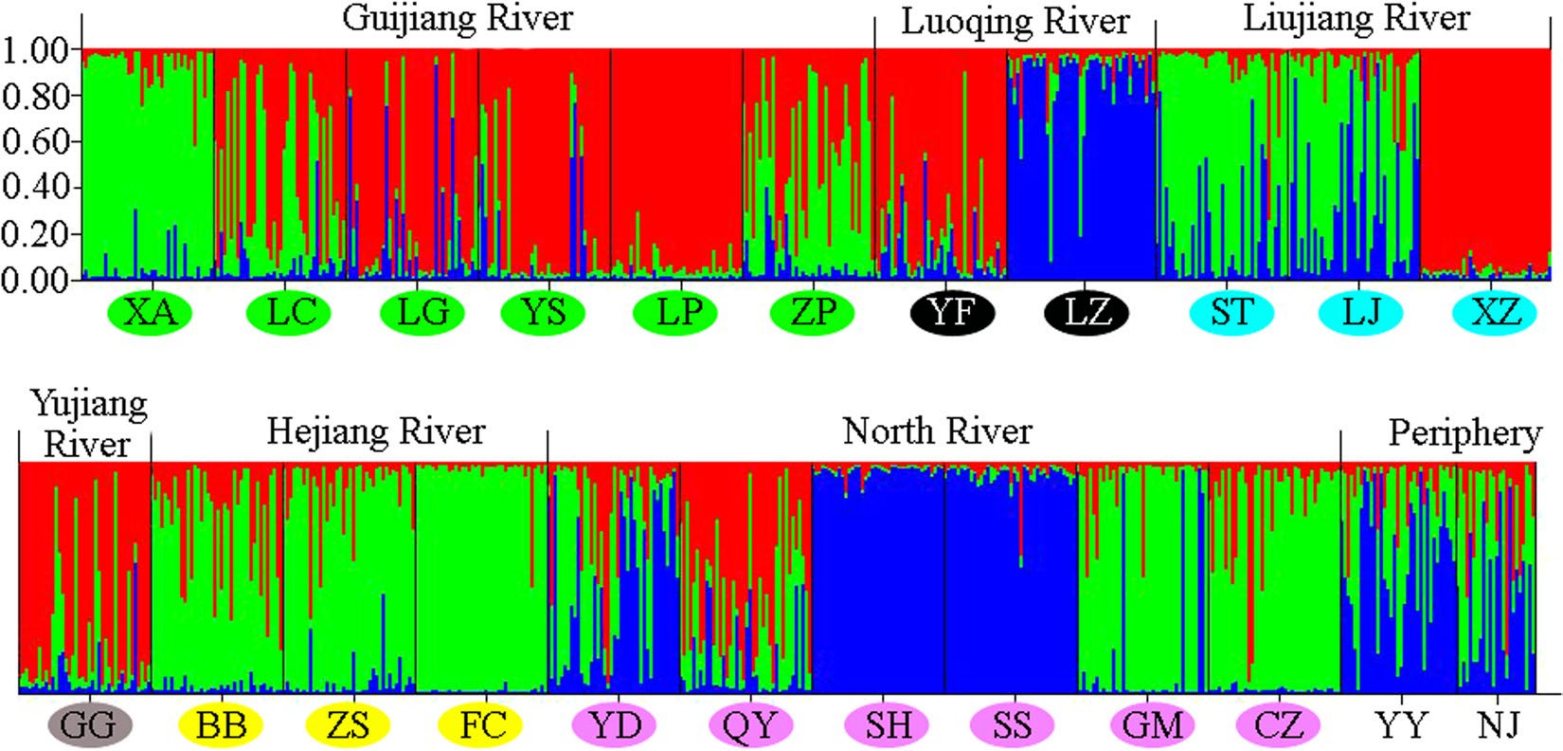
Bayesian results based on STRUCTURE among 23 populations of *P. clarkii* at *K* = 3; each individual was represented using a vertical bar displaying membership coefficients. Cluster 1 = red, cluster 2 = blue, cluster 3 = green. Populations with the same colour are distributed in a river. Lipu (LP), Lingui (LG), Yongfu (YF), Yangshuo (YS), Xing’an (XA), Lingchuan (LC), Shatang (ST), Luzhai (LZ), Liujiang (LJ), Xiangzhou (XZ), Guigang (GG), Babu (BB), Zhaoping (ZP), Zhongshan (ZS), Fuchuan (FC), Yingde (YD), Qingyuan (QY), Sihui (SH), Gaoming GM), Sanshui (SS), Chenzhou (CZ) Yueyang (YY) and Nanjing (NJ).

Based on our Bayesian assignment analysis, we determined *K* = 3 as the best model based on a plot of estimated log likelihood for the data at each *K* and a plot of the Δ*K* statistics (Figure S1). Structure analysis showed that the *P. clarkii* populations could be assigned to three distinct genetic clusters (Fig. 2). Six populations (LG, YS, LP, YF, XZ and GG) were mainly assigned as cluster 1 (red colour), three populations (LZ, SH and SS) were mainly assigned as cluster 2 (blue colour), and seven populations (XA, ST, BB, ZS, FC, GM and CZ) were mainly assigned as cluster 3 (green colour). The rest of the seven populations were mixed clusters. LC, ZP, and QY populations were mainly mixed by cluster 1 and cluster 3. Populations LJ, YD and YY were mainly mixed by cluster 2 and cluster 3. Only the population NJ was a mixed population consisting of all three clusters. At *K* = 3, some neighbouring populations (such as LZ and YF) within a river showed clear assignment to different clusters, whereas some geographically separated populations (such as LP and XZ) belonged to a single cluster and had substantial affinities.

**Figure 3:**
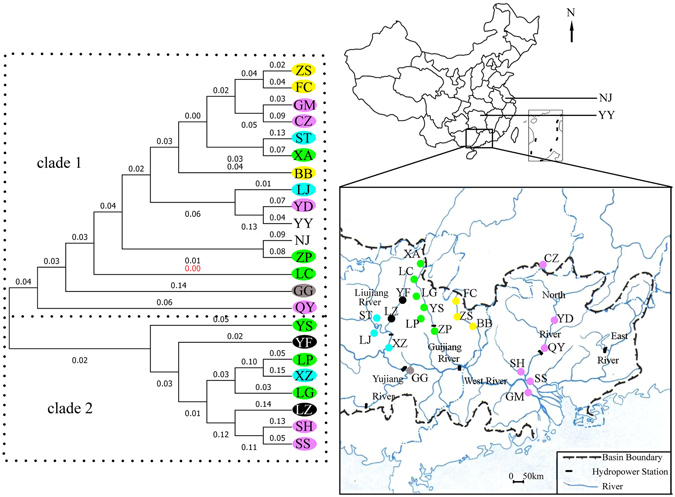
Neighbour-joining network based on Nei’s genetic distance for twenty-three populations of *P. clarkii*. Maps were obtained from the Pearl River Water Resources Commission^53^. Colour solid dots on maps indicate sample sites. Populations with the same colour are distributed in a river. Lipu (LP), Lingui (LG), Yongfu (YF), Yangshuo (YS), Xing’an (XA), Lingchuan (LC), Shatang (ST), Luzhai (LZ), Liujiang (LJ), Xiangzhou (XZ), Guigang (GG), Babu (BB), Zhaoping (ZP), Zhongshan (ZS), Fuchuan (FC), Yingde (YD), Qingyuan (QY), Sihui (SH), Gaoming GM), Sanshui (SS), Chenzhou (CZ) Yueyang (YY) and Nanjing (NJ).

The neighbour-joining tree based on Nei’s genetic distance recovers two largely distinct clades (Fig. 3). Populations YS, YF, LP, XZ, LG, LZ, SH and SS were gathered in one clade. The remaining populations were gathered in the other clade. Among the Guijiang River populations, populations XA, LC and ZP were gathered in clade 1, and the rest of populations YS, LG and LP were gathered in clade 2. Among the North River populations, populations CZ, YD, QY and GM were gathered in clade 1, and the rest of populations SS and SH were gathered in clade 2.

## Discussion

The genetic pattern observed in the Pearl River basin provides an illustration of human-mediated long-distance jump dispersal. Díezdelmolino *et al*. revealed high gene flow in river basins and similar genetic structures during the natural dispersal of mosquitofish (*Gambusia holbrooki*)^35^. If *P. clarkii* naturally spreads in rivers, the genetic structure will show large similarities, and there will be a correlation between geographic and genetic distance. Contrary to the hypothesis, some populations of *P. clarkii* along rivers had largely divergent genetic diversity (LG and XA, BB and FC, Table S1) and genetic structure (QY, SS and GM, Fig. 2). The IBD test also reflected no significant correlation between geographic and genetic distance in the Guijiang River and North River tributaries. We rule out a contiguous natural dispersal pathway in rivers. The best explanation may be that human activities were likely a major contributor to the long-distance dispersal of *P. clarkii*. Such anthropogenic migration of invasive species between distant sites will disrupt natural modes of dispersal and often result in limited genetic structure throughout an extended distribution^36^. Human-mediated dispersal is pervasive in other invasive freshwater species such as mosquitofish (*G. holbrooki*)^35^ and Chinese mitten crab (*Eriocheir sinensis*)^37^.

Ship-borne, attached to ship hulls and ballast water are expected as dispersal vectors for the long-distance dispersal of some non-native freshwater species^38^. Merchant shipping traffic was busy at midstream and downstream of the Pearl River basin. Greater shipping activity is expected to increase invasion success and genetic similarities at the population level by altering the diversity of discharged invasive species and the number of introduction events^39, 40^. However, merchant shipping did not reflect large genetic similarities in our study. The neighbour-joining tree showed a tangled pedigree. A large genetic distance even occurred among populations distributed in a river. Additionally, various types of hydropower station are distributed in the Pearl River basin. So we speculated shipping was improbable to mediated long-distance dispersal of *P. clarkii*. Land transportation (car, truck or train) was faster, more convenient and cost-effective than shipping. It might be the main vector to the dispersal of *P. clarkii*. This was a slightly different from dispersal vectors in the Yangtze River basin. Yangtze River basin is characterized by large basin area and many lakes. River drop is small and shipping is very busy and prosperous in midstream and downstream. Commercial shipping has likely facilitated communication among populations of *P. clarkii* in the Yangtze River basin and has influenced their population genetic structure and diversity^12, 21^. Both long-distance jump dispersals in the two River basins were explained by anthropogenic transportation.

Long-distance dispersal patterns are known to affect genetic structure between established populations, while also producing distinctive patterns of genetic differentiation during range expansion^41, 42^. There were seven genetic clusters in the Yangtze River basin^21^, but only three in the Pearl River basin. The observed genetic differentiation pattern within the Pearl River basin suggests multiple independent introductions from genetically divergent sources out of the Pearl River basin. Similar patterns were observed for invasive *Cordylophora* in the Great Lakes basin^4^. Notably, a low level of genetic differentiation was observed between populations YF and LG, indicating high levels of population connectivity and dispersal increase homogeneity. The homogeneity of the two geographically isolated populations further supported human-mediated dispersal.

Previous research noted that multiple introductions increase genetic variation and accelerate large-scale expansion of invasive species, which increase invasion success^43, 44^. Our results supported this view. The observed large genetic variation within populations suggested that some populations might have received multiple introductions. Even though some populations (YS, YF, LJ, QY, and YD) exhibited a harsh bottleneck effect, the genetic diversity was not obviously reduced. A founder effect is usually expected when a species, such as bullfrog (*Lithobates catesbeianaus*) and aphid (*Adelges cooleyi*), is introduced into a new habitat^45, 46^. However, founder effects and bottleneck effects do not always appear to be a barrier to successful invasion and subsequent expansion^47^. Multiple introductions from genetically divergent population might dilute the founder and bottleneck effects by rescuing some alleles^48^. The multiple introductions associated with long-distance dispersal of *P. clarkii* may have played important roles in shaping genetic variation, similar to other research^49, 50^.

Since the dispersal of *P. clarkii* is caused mainly by anthropogenic transportation, the primary management strategy to control *P. clarkii* should be control of anthropogenic transportation, such as the management of the common staring (*Sturnus vulgaris*)^10^ and bullfrogs^51^. Microsatellite evidence for the dispersal of invasive common starling provides information to produce a large-scale management strategy^10^. In 2005, the state of Montana designated bullfrogs (*L. catesbeianus*) as a prohibited species, which makes it illegal to possess, sell, purchase, exchange, or transport bullfrogs in Montana^51^. Since this legislation, management of which has been greatly effective in preventing bullfrogs from establishing a new population^51^. This study highlights the competence of genetic analysis to provide information for management of *P. clarkii*. Governments should create management policy to prevent dispersal. Prevention is clearly more cost-effective than post-establishment eradication or containment^5^. In our opinion, some simple management guidelines are: (1) governments enact constraint regulations on trade and introduction to prevent *P. clarkii* from escaping, or being released or cultivated and invest a large-scale education effort; (2) management agencies strictly enforce inspection at motor and railway stations in distribution area of *P. clarkii* to prevent transport. Deterring boaters from high-risk lakes is effective to reduce the spread of invasive species via human activities, and large-scale education efforts are more effective^52^.

Invasions are often complex and involve biotic and abiotic factors. Therefore, it is important to incorporate information to make comprehensive management strategies to ensure that sufficient effort is placed where required, thus maximizing the likelihood for successful control outcomes.

## Electronic supplementary material

Supplementary Information
