# Supplementary material for: Microsatellite evidence of dispersal mechanism of red swamp crayfish (Procambarus clarkii) in the Pearl River basin and implications for its management
Source: Sci Rep. 2017 Aug 15;7:8272. doi: 10.1038/s41598-017-08552-3 (PMC5557917; doi:10.1038/s41598-017-08552-3)
Supplement: Supplementary file 1 — Supplementary Information [file 41598_2017_8552_MOESM1_ESM.pdf]

Microsatellite evidence of dispersal mechanism of red swamp crayfish (*Procambarus clarkii*) in the Pearl River basin and implications for its management

Jinlong Huang, Shaoqing Tang, Fengjin Cai, Yanfang Lin, Zhengjun Wu*

Guangxi Key Laboratory of Rare and Endangered Animal Ecology, College of Life Science, Guangxi Normal University, Guilin, 541004, China

* Corresponding author: Zhengjun Wu

E-mail: wu_zhengjun@aliyun.com

Telephone: + 86 13517836091

Table S1. Location information and genetic diversity of *Procambarus clarkii* populations. *N*, number of individuals; *N*a, average number of alleles; *N*e, average number of effective alleles; *H*o, observed heterozygosity; *H*e, expected heterozygosity.

Site	Code	Collection	Latitude	Longitude	*N*	*N*a	*N*e	*H*o	*H*e
Guilin city									
Lingui district	LG	river	25°04′07.90″	110°12′45.61″	40	3.86	2.58	0.55	0.57
Yangshuo county	YS	pond	24°46′37.46″	110°29′04.63″	40	3.43	2.50	0.56	0.57
Yongfu county	YF	pond	24°59′09.93″	109°59′26.95″	40	3.43	2.44	0.56	0.56
Lingchuan county	LC	river	25°24′57.24″	110°19′14.25″	40	3.71	2.42	0.56	0.54
Lipu county	LP	ditch	24°29′51.21″	110°24′00.00″	40	2.71	1.89	0.49	0.44
Xing’an county	XA	ditch	25°36′46.17″	110°40′16.72″	40	3.29	1.98	0.38	0.43
Liuzhou city									
Liujiang county	LJ	pond	24°14′55.56″	109°21′55.79″	40	3.00	2.22	0.48	0.56
Shatang town	ST	pond	24°27′13.89″	109°22′42.85″	40	3.29	2.18	0.34	0.51
Luzhai county	LZ	river	24°24′10.73″	109°36′25.22″	45	3.14	2.09	0.46	0.43
Laibin city									
Xiangzhou county	XZ	river	23°58′02.02″	109°41′33.15″	40	2.86	1.75	0.40	0.39
Guigang city	GG	pond	23°05′39.03″	109°38′06.98″	40	2.57	2.06	0.46	0.47
Hezhou city									
Babu district	BB	ditch	24°24′57.21″	111°34′38.19″	40	3.29	2.24	0.53	0.51
Zhaoping county	ZP	river	24°10′52.34″	110°48′19.26″	40	3.00	2.05	0.46	0.48
Zhongshan county	ZS	pond	24°31′39.42″	111°18′00.93″	40	3.29	2.18	0.46	0.46
Fuchuan county	FC	river	24°49′18.33″	111°16′16.78″	40	2.57	1.73	0.25	0.29
Yingde city	YD	pond	24°10′50.16″	113°24′41.69″	40	3.00	2.51	0.44	0.56
Qingyuan city	QY	pond	23°41′08.24″	113°03′04.56″	40	3.71	2.65	0.60	0.58
Sihui city	SH	pond	23°20′23.29″	112°41′39.59″	40	4.00	2.75	0.56	0.59
Foshan city									
Gaoming district	GM	pond	22°53′24.76″	112°52′27.91″	40	4.00	2.23	0.39	0.48
Sanshui district	SS	river	23°09′33.02″	112°53′39.32″	40	3.57	2.57	0.56	0.58
Chenzhou city	CZ	river	25°46′15.78″	113°00′55.88″	40	2.57	1.87	0.40	0.40
Yueyang city	YY	river	29°32′07.01″	112°44′33.60″	35	4.14	2.63	0.54	0.59
Nanjing city	NJ	river	32°03′38.40″	118°47′44.76″	24	3.71	2.69	0.60	0.60

Table S2. Pairwise F_{ST} values of twenty-three populations are shown below the diagonal.

	LP	LG	YF	YS	XA	LC	ST	LZ	LJ	XZ	GG	BB	ZP	ZS	FC	YD	QY	SH	GM	SS	CZ	YY	NJ
LP	0.00																						
LG	0.10	0.00																					
YF	0.11	0.01	0.00																				
YS	0.07	0.04	0.04	0.00																			
XA	0.19	0.14	0.11	0.14	0.00																		
LC	0.15	0.07	0.06	0.06	0.08	0.00																	
ST	0.17	0.14	0.11	0.13	0.09	0.11	0.00																
LZ	0.18	0.11	0.13	0.16	0.20	0.18	0.20	0.00															
LJ	0.19	0.11	0.08	0.10	0.08	0.05	0.07	0.16	0.00														
XZ	0.10	0.08	0.11	0.12	0.25	0.16	0.26	0.16	0.23	0.00													
GG	0.19	0.13	0.13	0.11	0.20	0.06	0.18	0.25	0.14	0.21	0.00												
BB	0.14	0.11	0.09	0.07	0.09	0.05	0.07	0.22	0.06	0.23	0.12	0.00											
ZP	0.19	0.08	0.07	0.11	0.08	0.05	0.11	0.19	0.07	0.16	0.15	0.07	0.00										
ZS	0.21	0.19	0.16	0.13	0.12	0.07	0.11	0.26	0.08	0.31	0.09	0.06	0.14	0.00									
FC	0.31	0.28	0.25	0.21	0.15	0.13	0.16	0.35	0.13	0.41	0.21	0.10	0.17	0.05	0.00								
YD	0.19	0.12	0.10	0.10	0.16	0.10	0.10	0.22	0.07	0.23	0.14	0.09	0.13	0.11	0.20	0.00							
QY	0.06	0.07	0.08	0.04	0.14	0.06	0.11	0.16	0.10	0.12	0.10	0.06	0.11	0.11	0.19	0.10	0.00						
SH	0.17	0.09	0.10	0.12	0.20	0.15	0.18	0.09	0.14	0.16	0.19	0.19	0.16	0.22	0.33	0.15	0.13	0.00					
GM	0.19	0.16	0.14	0.13	0.08	0.08	0.07	0.25	0.07	0.29	0.17	0.06	0.11	0.07	0.08	0.11	0.12	0.21	0.00				
SS	0.19	0.10	0.09	0.11	0.18	0.13	0.15	0.16	0.12	0.22	0.17	0.13	0.14	0.18	0.28	0.12	0.12	0.05	0.17	0.00			
CZ	0.20	0.13	0.14	0.14	0.11	0.09	0.14	0.21	0.12	0.23	0.22	0.10	0.10	0.14	0.15	0.18	0.14	0.22	0.07	0.22	0.00		
YY	0.19	0.11	0.10	0.10	0.15	0.07	0.10	0.20	0.06	0.21	0.12	0.10	0.12	0.10	0.18	0.03	0.10	0.13	0.10	0.10	0.15	0.00	
NJ	0.19	0.09	0.09	0.10	0.11	0.06	0.10	0.15	0.07	0.20	0.10	0.07	0.07	0.08	0.15	0.09	0.08	0.12	0.10	0.10	0.12	0.08	0.00

Location abbreviations are defined in Table 1

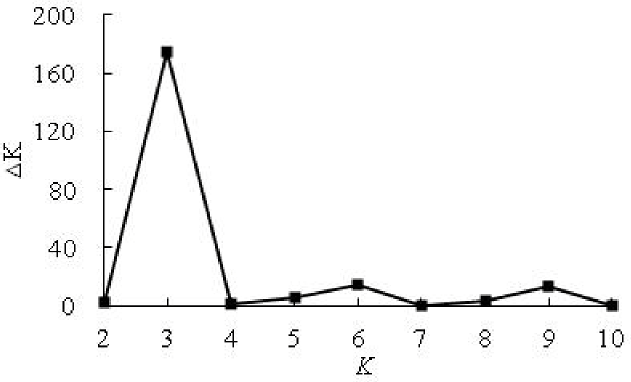

Figure S1. Plot of ΔK vs. K analyzed in Bayesian inference of population structure.
